# Analysis of Changes in Plasma Cytokine Levels in Response to IL12 Therapy in Three Clinical Trials

**DOI:** 10.1158/2767-9764.CRC-23-0122

**Published:** 2024-01-10

**Authors:** Emily Schwarz, Brooke Benner, Lianbo Yu, Fode Tounkara, William E. Carson

**Affiliations:** 1 Biomedical Sciences Graduate Program, College of Medicine, The Ohio State University, Columbus, Ohio.; 2 Comprehensive Cancer Center, The Ohio State University, Columbus, Ohio.; 3 Center for Biostatistics, The Ohio State University Wexner Medical Center, Columbus, Ohio.; 4 Department of Biomedical Informatics, The Ohio State University, Columbus, Ohio.; 5 Department of Surgery, Division of Surgical Oncology, The Ohio State University, Columbus, Ohio.

## Abstract

The ability of IL12 to stimulate natural killer (NK) cell and T-cell antitumor activity makes it an attractive candidate for the immune therapy of cancer. Our group has demonstrated that IL12 enhances the NK cell response to antibody-coated tumor cells and conducted three clinical trials utilizing IL12 with mAbs (OSU-9968, OSU-0167, and OSU-11010). To better characterize IL12-induced immunity, plasma cytokine levels were measured in 21 patients from these trials with favorable and unfavorable responses. *t*-statistics and linear modeling were used to test for differences within and between response groups by examining levels at baseline and post-IL12 administration. Patients exhibited significant increases in 11 cytokines post-IL12 administration when analyzed collectively. However, several cytokines were differentially induced by IL12 depending on response. GMCSF was significantly increased in complete/partially responding patients, while stable disease patients had significant increases in IL10 and decreases in VEGF-C. Patients who experienced progressive disease had significant increases in CCL3, CCL4, IL18, TNFα, CXCL10, CCL8, CCL2, IL6, and IFNγ. The increases in CCL3, CCL4, and IL6 in progressive disease patients were significantly higher than in clinically benefitting patients and most prominent within the first two cycles of IL12 therapy. This correlative pilot study has identified changes that occur in levels of circulating cytokines following IL12 administration to patients with cancer, but this report must be viewed as exploratory in nature. It is meant to spark further inquiry into the topic via the analysis of additional cohorts of patients with similar characteristics who have received IL12 in a uniform fashion.

**Significance:**

IL12 activates immune cells and is used to treat cancer. The profile of circulating cytokines was measured in an exploratory fashion in patients with cancer that received IL12 in combination with mAbs. This correlative pilot study could serve as the basis for additional studies of IL12 effects on the production of immune cytokines.

## Introduction

IL12 is a proinflammatory cytokine capable of stimulating both innate and adaptive immune responses (1). It can be produced endogenously by several cell types including macrophages, monocytes, dendritic cells, B cells, and neutrophils (2). IL12 signaling occurs via activation of the IL12 receptor (IL12R) which is primarily expressed on activated natural killer (NK) and T cells, but has also been found on dendritic cells and B cell lines (3, 4). The IL12R contains two type I transmembrane glycoprotein chains; IL12Rβ1 and IL12Rβ2. Signal transduction is mediated via the IL12Rβ2 which induces phosphorylation of Janus kinase (JAK)-signal transducer and activator of transcription (STAT) proteins downstream (5). IL12R activation results in the phosphorylation of JAK2 and TYK2, which then phosphorylate STAT1, STAT3, STAT4, and STAT5 (2, 6). This signaling cascade results in several downstream effects such as enhanced IFNγ production by NK cells and T cells in response to STAT4 activation. IFNγ can in turn activate macrophages, NK cells and B cells, as well as promote a Th1 T-cell response through T-bet upregulation (7). The ability of IL12 to stimulate NK cells and T cells has made it an attractive candidate for inducing antitumor responses in a wide variety of cancer types (8–10).

The first clinical trials utilizing IL12 for the treatment of cancer began in the 1990s after several successful preclinical models showed that it had antitumor activity. However, early toxicity issues led to a temporary cease of its use in 1995 (11). A follow-up investigation by Leonard and colleagues determined that the toxicity resulted from induction of IFNγ in response to high doses, but a test dose of IL12 prior to high-dose treatment could abrogate this toxicity (12). As a result of an improved understanding of IL12 biology and the effect of alternate treatment schedules, there has been a resurgence in its therapeutic use (13). Three clinical trials that capitalized on the renewed availability of IL12 for cancer therapy were conducted by our group: OSU-9968, OSU-0167, and OSU-11010. OSU-9968 was a phase I trial of IL12 and trastuzumab conducted at The Ohio State University (Columbus, OH) from 1999 to 2003 in patients with HER2-expressing non-hematologic malignancies (14). OSU-0167 was a follow-up phase I trial of IL12 in combination with trastuzumab and paclitaxel conducted at The Ohio State University (Columbus, OH) between 2002 and 2004 enrolling patients with HER2-expressing non-hematologic malignancies (15). OSU-11010 was a multisite phase I/II clinical trial of IL12 in combination with cetuximab open between 2014 and 2015 to patients with recurrent and/or metastatic head and neck squamous cell carcinoma (16). Clinical responses and prolonged stabilization of disease were seen in these studies (14–16). These studies demonstrated that lower doses of IL12 could have anticancer activity. These results highlight the need for additional understanding of the mechanism of patient responses to IL12.

Many studies have attempted to correlate biological analytes with patient response to IL12 therapy. In a comprehensive review, it was found that increased levels of IFNγ, CXCL10, TNFα, CCL3, CXCL9, and CD4^+^ and CD8^+^ T cells, and decreased levels of VEGF, bFGF, FoxP3^+^ regulatory T cells, and M2 macrophages were found to correlate with favorable patient responses (17). The current study sought to further investigate potential biomarkers of patient responses to IL12 therapy by analyzing shared changes in circulating cytokines levels before and after IL12 treatment in a subset of patients across multiple studies (OSU-9968, OSU-0167, OSU-11010). Patients were defined as having experienced progressive disease (PD), stable disease (SD), or complete/partial responses (CR/PR) and their corresponding changes in cytokine profiles over time were evaluated in hopes of identifying potential biomarkers of responses to IL12 therapy. The results of this correlative pilot study should be viewed as exploratory in nature due to the heterogeneity of the patient populations and treatments and the coadministration of antitumor mAbs in all three studies.

## Materials and Methods

### Overview of OSU-9968, OSU-0167, and OSU-11010

OSU-9968 is a NCI (T99-0032) sponsored phase I trial of IL12 in combination with trastuzumab that was conducted at The Ohio State University Comprehensive Cancer Center between August 1999 and January 2003 (14). A total of 15 patients were enrolled with HER2-expressing non-hematologic malignancies including only those ≥18 years of age with a life expectancy >6 months and normal bone marrow and organ function. Additional inclusion and exclusion criteria were published previously (14). Patient ages ranged from 32 to 71 with a median of 52 and a 14:1 ratio of females to males. One patient on this study experienced a CR, 2 had significant disease stabilization, and 12 experienced disease progression. Plasma samples from 8 patients were included in this current study under approval from the Human Institutional Review Board (IRB) at The Ohio State University Medical Center (Columbus, OH; 99H0185). OSU-0167 is NCI (84) sponsored phase I trial of IL12 in combination with trastuzumab and paclitaxel conducted at The Ohio State University Comprehensive Cancer Center between January 2002 and July 2004 (15). A total of 21 patients including 11 males and 10 females with HER2-overexpressing non-hematologic malignancies were included in this trial. Exclusion criteria included any patients who had previous therapy with trastuzumab. Additional inclusion and exclusion criteria were published previously (15). Patient ages ranged from 41 to 81 with a median of 56. One patient experienced a CR, 4 had PRs, 6 had disease stabilization lasting at least 3 months, and 10 had disease progression. Plasma samples from 5 patients were included in this current study with IRB approval (1999C0326). OSU-11010 is a NCI (8860) sponsored phase I/II trial of IL12 in combination with cetuximab which was conducted between April 2014 and July 2015 (16). This was a multisite clinical trial with The Ohio State University Comprehensive Cancer Center serving as the lead site. Total 23 patients including 22 males and 1 female were enrolled with recurrent and/or metastatic head and neck squamous cell carcinoma. Further inclusion and exclusion criteria were published previously (16). Twenty-two patients were evaluable for response at the conclusion of this study with 16 experiencing disease stabilization (some marked) and 6 experiencing disease progression. Plasma samples from 8 patients were included in this current study with IRB approval (2011C0019). Attrition was low in all three trials with only one patient removed from OSU-11010 due to changes in liver enzymes. The evidence for treatment effect in SD patients was based on an observation of clinical benefit (CB) wherein the patient's tumor growth was perceptibly slowed upon the institution of therapy. Randomization and blinding techniques were not applicable due to the phase I status of each of the three trials. In total, 67 banked plasma samples from 21 patients enrolled in these three trials were utilized for this study with samples from 9 PD patients, 9 SD patients (progression-free disease >60 days), 1 PR, and 2 CR patients included. Plasma samples were isolated from peripheral blood draws at baseline and 72-hour post-IL12 administration throughout treatment.

### Treatment Schemas

In OSU-9968, a baseline peripheral blood draw was collected on day 1 prior to the start of treatment. Patients were then given an intravenous loading dose of trastuzumab at 4 mg/kg which was followed by a weekly maintenance dose of 2 mg/kg. Trastuzumab was given on day 1 of each week-long cycle. Starting with week 3, patients were given an intravenous dose of IL12 on day 2 and day 5 of the weekly cycle. A post-IL12 blood draw was performed just prior to the day 5 IL12 administration. This represented the 72-hour post-IL12 timepoint used in the current study. No other treatments were given between the day 2 and day 5 doses of IL12. The day 2 dose of IL12 also occurred at least 24 hours after trastuzumab administration. In OSU-0167, the same baseline peripheral blood draw was collected prior to treatment. On day 1 of the 3-week cycle, patients received a 4 mg/kg loading dose of trastuzumab intravenously (lowered to 2 mg/kg on day 1 of weeks 2 and 3), followed by a 175 mg/m^2^ dose of paclitaxel. Paclitaxel was given only once every 3-week cycle. Beginning with cycle 2, IL12 was given by intravenous on days 2 and 5 of each week. IL12 was given 24 hours after trastuzumab/paclitaxel or trastuzumab treatment. As with OSU-9968, a post-IL12 timepoint as was collected just prior to IL12 treatment on day 5 of each week. Only IL12 was administered in the 72 hours between blood draws. OSU-11010 also had a baseline peripheral blood draw collected prior to the start of treatment. Treatment cycles were 2 weeks long with cetuximab being administered intravenously at a dose of 500 mg/m^2^ on day 1 of the cycle. Beginning with cycle 2, IL12 treatment was given by subcutaneous injection on days 2 and 5 of the cycle. The first injection of IL12 was given 24 hours following cetuximab treatment. The post-IL12 timepoint was collected 72 hours after the day 2 dose of IL12 (prior to treatment on day 5). Just as in OSU-9968 and OSU-0167, no additional treatments were administered between the day 2 and day 5 dose of IL12. In total, 17 of the 21 patients (all patients from OSU-0167 and OSU-11010) received systemic IL12 therapy at a dose of 300 ng/kg. Two patients in OSU-9968 received a 100 ng/kg dose of systemic IL12 and 2 patients received a 500 ng/kg dose. Detailed treatment schemas for each trial are depicted in Table 1 for further reference.

**TABLE 1:** Treatment schemas for OSU-0167, OSU-9968, and OSU-11010

OSU-0167: Phase I study of IL12 + trastuzumab + paclitaxel, Cycle 2 and Up:								
Week 1	Day:	1	2	3	4	5	6	7
Treatment		t/P	12			12		
Week 2		8	9	10	11	12	13	14
Treatment		t	12			12		
Weeks 3		15	16	17	18	19	20	21
Treatment		t	12			12		
(t) Trastuzumab (2mg/kg i.v.), (P) Paclitaxel (175 mg/m^2^ i.v.), (12) IL12 (300 ng/kg i.v.)								
**OSU-9968: Phase I study of IL12 + trastuzumab, Cycle 1 and Up:**								
Week 1	Day:	1	2	3	4	5	6	7
Treatment		T						
Week 2		8	9	10	11	12	13	14
Treatment		t						
Weeks 3–54		15	16	17	18	19	20	21
Treatment		t	12			12		
(T) Trastuzumab (4 mg/kg i.v.), (t) Trastuzumab (2mg/kg i.v.), (12) IL12 (100, 300, 500 ng/kg i.v.)								
**OSU-11010: Phase I study of IL12 + cetuximab, Cycle 2 and Up:**								
Week 1	Day:	1	2	3	4	5	6	7
Treatment		C	12			12		
Week 2		8	9	10	11	12	13	14
Treatment								
(C) Cetuximab (500 mg/m^2^ i.v.), (12) IL12 (200 ng/kg or 300 ng/kg s.c.)								

Abbreviations: i.v., intravenous; s.c., subcutaneous.

### Plasma Sample Collection

Approximately 20 mL of peripheral blood was collected in green-top heparinized tubes pretreatment (baseline) and just prior to each therapeutic administration of IL12 or respective antibodies/chemotherapy in all three studies. Following collection, tubes were centrifuged at 1,700 rpm for 10 minutes after which plasma was procured and stored at −80˚C until further use. In total, 67 plasma samples from 21 patients enrolled in OSU-9968, OSU-0167, and OSU-11010 were utilized for this study. Samples were included from 9 PD patients, 9 SD patients (progression-free disease >60 days), 1 PR, and 2 CR patients. Timepoints in which cytokine levels were assayed included baseline, C2D5 (cycle 2 day 5), C2D8, C2D9, C2D12, C2D15, C2D19, C3D1, C4D5, C5D1, C5D2, C5D5, C6D1, C6D5, and C12D5. Post-IL12 timepoints for each patient included the earliest plasma sample available 72 hours following IL12 administration.

### Measurement of Plasma Cytokine Levels

Cytokine levels in 67 patient plasma samples were measured using seven different 96-well custom electrochemiluminescence-based U-PLEX, R-PLEX, and V-PLEX human biomarker assays as per manufacturer instructions (Meso Scale Discovery, LLC). Four custom-made U-plex human biomarker assays were used to measure IFNγ, GMCSF, TNFα, IL1α, IL1ß, IL4, IL5, IL6, IL8, IL10, IL13, IL15, IL17, IL18, CXCL10, CCL2, CCL8, CCL22, CCL3, and CCL4 (Meso Scale Discovery, catalog nos. K15067M-1 and K15067L-1) in a blinded fashion. One additional U-plex human biomarker assay was used to measure TGFß (Meso Scale Discovery, catalog no. K151XWK-1). One V-plex human biomarker assay was used to measure VEGF-C (Meso Scale Discovery, #K151A9H-1) and one R-plex human biomarker assay was used to measure CXCL9 levels (Meso Scale Discovery, catalog no. F2101-3). Briefly, biotinylated capture antibodies were coupled to proprietary linkers by vortexing and incubating for 30 minutes at room temperature. A total of 200 µL of stop solution was then added to each linker-coupled antibody solution, with the solution then vortexed and brought up to 6 mL by adding additional stop solution. A total of 50 µL of this final solution was then added to each well in the multiplex assay to coat the entire plate. After shaking the plate for 1 hour at room temperature, the plate was washed three times with 150 µL of PBS with 0.05% Tween-20 (PBS-T). A total of 25 µL of diluent 43 was then added to each well in the plate. Next, 25 µL of either calibrator standards or undiluted plasma samples were added to each well. All samples were added in duplicates using a multichannel pipettor with concentrations averaged following plate reading to reduce intrasample variation. The plate was then sealed and incubated while shaking for 1 hour at room temperature. After incubating, the plate was once again washed three times with 150 µL PBS-T per well. A total of 50 µL of SULFO-TAG conjugated detection antibody was then added to each well and the plate was seal and incubated with shaking again for 1 hour at room temperature. After 1 hour, 150 µL per well of PBS-T was used to wash the plate three separate times. A total of 150 µL Read Buffer T was then added to each well and the plate was read immediately using a Quickplex SQ 120 MSD plate reader in the Clinical Research Center Analytical and Development Laboratory at The Ohio State University. MSD Discovery Workbench software version 4.0 was utilized to generate standard curves and measure cytokine concentrations in each sample.

### Statistical Analysis

Paired *t* tests were used to determine whether there were significant changes in patient cytokine levels after IL12 administration compared with baseline levels. Linear modeling and *t*-statistics were used to test differences in cytokine profile changes within patient groups with Bonferroni method applied *post hoc* to adjust for multiple comparisons. ANOVA was then used to test whether differences in cytokine profile changes between response groups were statistically significant. For each comparison, an observed difference was declared to be significant when the *P* value of the test was smaller than the significant level which was fixed at 0.05. Cytokine levels are presented as mean ± SD for intragroup analyses and mean ± SE for intergroup analyses.

### Data Availability

The data generated in this study are currently available upon request from the corresponding author.

## Results

### IL12 Induces Significant Changes in Circulating Cytokine and Chemokine Levels

**FIGURE 1:**
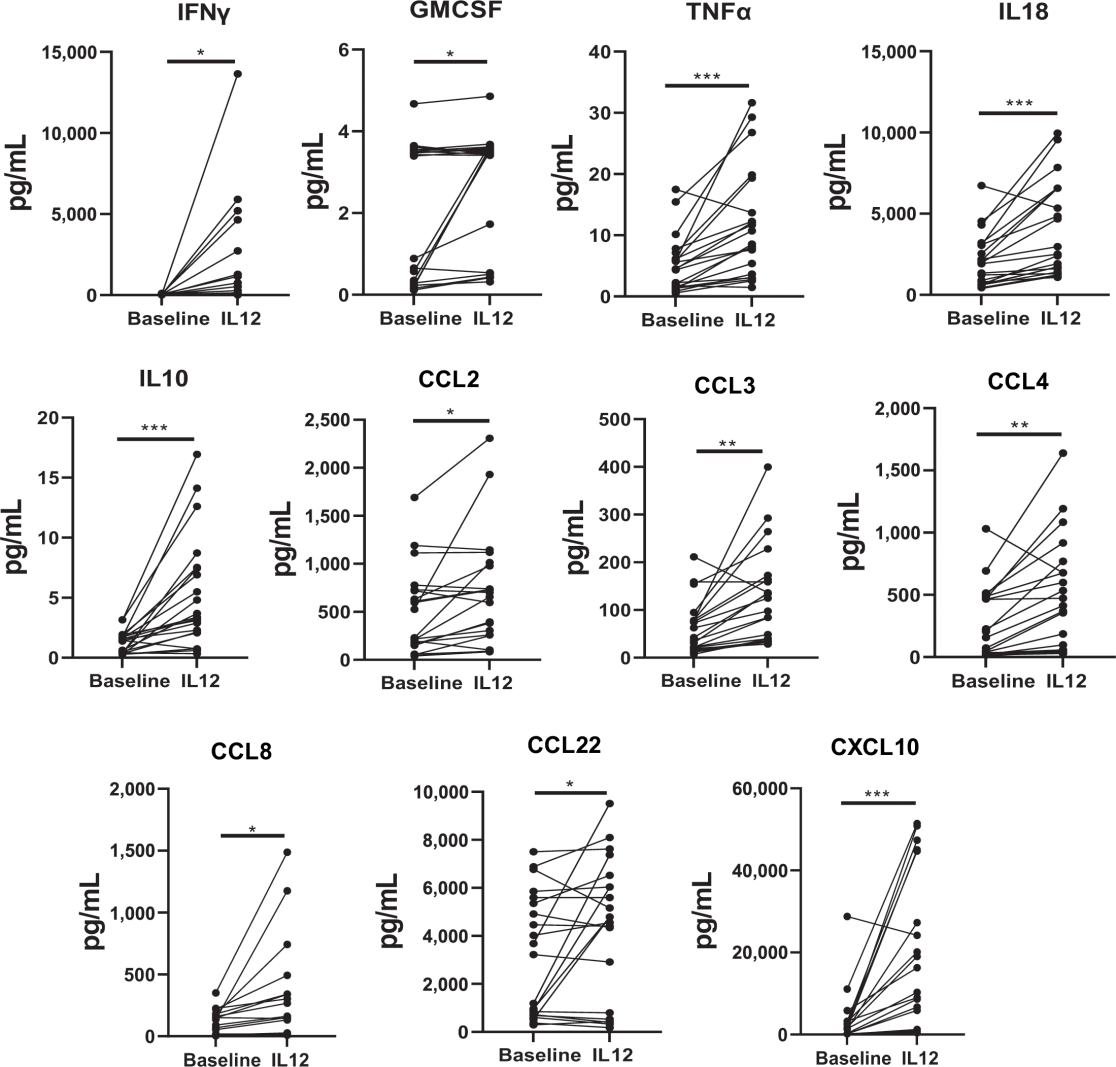
Plasma cytokine levels from all patients (*n* = 21) measured at baseline and 72-hour post-IL12 treatment. Patient plasma samples collected 72 hours after the first, second, or third dose of IL12 were measured for levels of 23 cytokines (pg/mL). Samples were run in triplicate using seven multiplex assays. The average of the triplicates was plotted for each patient, represented by a single dot. Results shown represent the nine cytokines found to have significantly upregulated mean levels following IL12 administration. *P* = 0.02 for IFNγ, *P* = 0.02 for GMCSF, *P* < 0.001 for TNFα, *P* < 0.001 for IL18, *P* < 0.001 for IL10, *P* = 0.01 for CCL2, *P* = 0.001 for CCL3, *P* = 0.002 for CCL4, *P* = 0.02 for CCL8, *P* = 0.03 for CCL22, and *P* < 0.001 for CXCL10. Two-sided paired *t* tests, *, *P* < 0.05; **, *P* < 0.01; ***, *P* < 0.001.

The circulating levels of 23 cytokines and chemokines (GMCSF, IFNγ, IL10, IL8, CXCL10, CCL2, CCL22, CCL3, CCL4, TNFα, IL15, IL18, CCL8, CXCL9, IL13, IL17, IL1ß, IL4, IL5, IL6, IL1α, TGFβ, VEGF-C) that are highly involved in orchestrating the immune response to cancer were measured in the plasma of patients (*n* = 21) who received IL12 in three different clinical trials; OSU-9968, OSU-0167, or OSU-11010 and experienced either a PR or CR clinical response, SD or PD following administration of this cytokine (Tables 1 and 2). When this group of patients was evaluated as a whole, 11 cytokines and chemokines were found to be significantly increased 72 hours post-IL12 administration compared with baseline levels (Fig. 1). These factors included proinflammatory cytokines known to be produced by IL12-stimulated NK cells such as IFNγ (*P* = 0.02), GMCSF (*P* = 0.02), and TNFα (*P* < 0.001). IL18 was also significantly increased (*P* < 0.001) following IL12 administration. IL10 is considered to be anti-inflammatory, and its levels were also significantly increased after IL12 treatment (*P* < 0.001). Finally, several chemokines associated with monocyte, dendritic cell, macrophage, and T-cell migration were found to be significantly upregulated following IL12 treatment including CCL3 (*P* = 0.001), CCL4 (*P* = 0.002), CCL2 (*P* = 0.01), CCL8 (*P* = 0.02), CCL22 (*P* = 0.03), and CXCL10 (*P* < 0.001). Levels of the remaining cytokines (IL8, IL15, MIG, IL13, IL17, IL1ß, IL4, IL5, IL6, IL1α, TGFβ, VEGF-C) did not change significantly with IL12 administration when the group as a whole was evaluated.

**TABLE 2:** Tumor type, treatment, and clinical outcomes of patients (*n* = 21) included from OSU-0167, OSU-9968, and OSU-11010

OSU-0167: Phase I study of IL12 + trastuzumab + paclitaxel					
Patient	Tumor type (HER2 level)	IL12 Dose (ng/kg)	IL12 administration	Clinical outcome	Cycles given
E	Breast (3+)	300	i.v.	PD	2
F	Colon (2+)	300	i.v.	PD	3
G	Breast (3+)	300	i.v.	PR	3
H	Breast (2+)	300	i.v.	SD	6
I	Esoph. (2+)	300	i.v.	PR	6
**OSU-9968: Phase I study of IL12 + trastuzumab**					
**Patient**	**Tumor type (HER2 level)**	**IL12 Dose (ng/kg)**	**IL12 Administration**	**Clinical outcome**	**Cycles given**
E	Breast (3+)	100	i.v.	CR	54
F	Breast (2+)	100	i.v.	PD	14
H	Breast (2+)	300	i.v.	PD	14
K	Breast (2+)	300	i.v.	PD	10
L	Breast (3+)	500	i.v.	PD	14
M	Breast (3+)	500	i.v.	SD	34
P	Breast (3+)	300	i.v.	PD	14
Q	Breast (2+)	300	i.v.	SD	54
**OSU-11010: Phase I study of IL12 + cetuximab**					
**Patient**	**Tumor type (HER2 level)**	**IL12 Dose (ng/kg)**	**IL12 Administration**	**Clinical outcome**	**Cycles given**
I	HNSCC	300	s.c.	PD	4
K	HNSCC	300	s.c.	SD	33
L	HNSCC	300	s.c.	SD	32
M	HNSCC	300	s.c.	SD	32
R	HNSCC	300	s.c.	PD	4
T	HNSCC	300	s.c.	PD	4
V	HNSCC	300	s.c.	SD	19
W	HNSCC	300	s.c.	SD	4

Abbreviations: HNSCC, head and neck squamous cell carcinoma; i.v., intravenous.

### Overall Trends in Cytokine/Chemokine Profiles Following IL12 Therapy are Distinct Between Patient Response Groups

**FIGURE 2:**
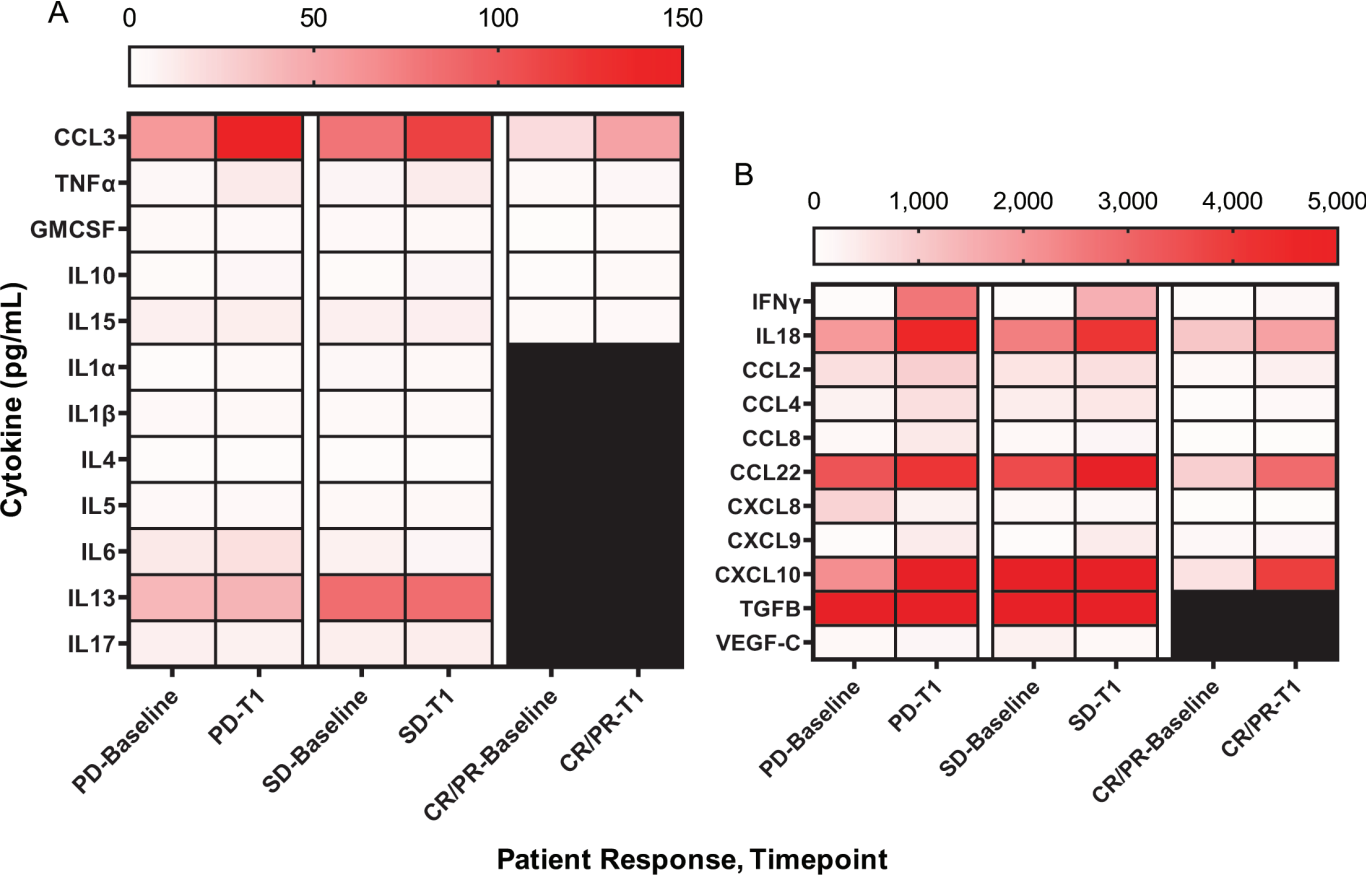
Heat map showing patient (*n* = 21) plasma levels of 23 cytokines ranging from 0 to 150 pg/mL in **A** and 0 to 5,000 pg/mL in **B**. The presence of red coloring represents increasing levels of each cytokine over 0 pg/mL, which is depicted by the color white. Individual patients are categorized by time and response, with the first two columns representing baseline and post-IL12 timepoints of PD patients, the second two columns representing baseline and post-IL12 timepoints of SD patients and the third two columns representing baseline and post-IL12 timepoints of CR/PR patients. Each of the 23 cytokines analyzed are labeled on the *y*-axis. Averages from each respective subset of patients included are plotted and were measured by multiplex assays. The color gray represents samples unavailable for inclusion.

We next explored the changes in cytokine/chemokine profiles in response to IL12 therapy when patients were grouped according to how they responded to therapy. We divided patients into three response groups: CRs/PRs (*n* = 3), SD patients (*n* = 9), and PD patients (*n* = 9). The patterns of cytokine/chemokine fluctuation within each patient response group are presented graphically in the heat map in Fig. 2. IFNγ, CCL3, and IL18 were induced in all three groups. However, changes from baseline levels to the first timepoint collected 72 hours post-IL12 administration were notably different for several cytokines and chemokines according to response group. CCL8 was present at high levels in the circulation at baseline and increased following IL12 therapy in both PD (119.54 ± 120.46 to 434.12 ± 544.92 pg/mL) and SD patients (105.70 ± 76.68 to 214.22 ± 220.67 pg/mL). In contrast, levels of this factor at the two timepoints were much lower in CR and PR patients and did not respond to IL12 (10.77 ± 7.99 to 15.62 ± 3.30 pg/mL). Showing a somewhat similar pattern, CXCL9 levels were low at baseline in PD and SD patients and then rose following the administration of IL12 (26.60 ± 6.99 to 409.88 ± 604.06 pg/mL and 30.04 ± 8.89 to 395.93 ± 117.23 pg/mL, respectively), whereas levels in responders were relatively high at baseline and remained at that level (145.05 ± 200.00 to 178.15 ± 40.50 pg/mL). CXCL10 increased in all response groups following IL12 administration but was markedly higher in PD patients (2192.83 ± 3483.81 to 230002.01 ± 22073 pg/mL, or 10.49-fold). The chemokine CCL22 was present at very high levels at baseline in PD (3372.91 ± 2749.13 pg/mL) and SD patients (3580.01 ± 2563.46 pg/mL) but were not as high in CR/PR patients (918.59 ± 243.02 pg/mL). Mirroring this pattern, levels of IL8 were measurable at baseline in PD patients but very low in the other groups. CCL4 was similarly expressed at higher levels in progressive and SD; however, absolute increases were seen in each group upon receipt of IL12. The increases in CCL4 for PD patients were higher (239.67 ± 250.79 and 621.02 ± 581.53 pg/mL, *P* = 0.001) than those for SD patients (365.76 ± 325.79 and 486.97 ± 282.25) and CR/PR patients (25.71 ± 12.53 and 87.57 ± 86.20 pg/mL). Thus, responders were characterized by low levels of CCL8 at the two timepoints, high and unchanging levels of CXCL9 and inducible but overall lower levels of CCL22 and CCL4.

### Statistical Analysis of Changes in Cytokine/Chemokine Levels After IL12 Therapy Reveals Unique Differences Depending on Patient Response

We applied statistical modeling to analyze specific changes in levels of each circulating cytokine and chemokine measured from baseline to the first timepoint within the three patient response groups (CR/PR, SD, or PD). We found that each patient response group had a unique set of cytokines which were significantly induced in response to treatment with IL12 (Table 3). GMCSF was the only cytokine significantly induced in CR/PR patients with an average increase of 1.45 pg/mL 72-hour post-IL12 administration (*P* = 0.05). Neither SD nor PD patients had a significant increase in GMCSF in response to IL12. Rather, SD patients exhibited a significant increase in IL10 (3.63 pg/mL, *P* = 0.02) along with a significant decrease in the angiogenic factor VEGF-C (103.31 pg/mL, *P* = 0.05). PD patients also had a significant increase in IL10 (3.75 pg/mL, *P* = 0.02), but shared no other changes with SD or CR/PR patients. PD patients instead had significant increases in nine other cytokines including CCL3 (*P* < 0.001), CCL4 (*P* = 0.001), IL18 (*P* = 0.003), TNFα (*P* = 0.004), CXCL10 (*P* = 0.005), CCL8 (*P* = 0.01), CCL2 (*P* = 0.03), IL6 (*P* = 0.03), and IFNγ (*P* = 0.04).

**TABLE 3:** Statistical analysis of cytokine profile changes after IL12 treatment within patient response groups. Cytokine profiles consisting of 23 different cytokines were analyzed for significant changes between baseline and 72 hours post-IL12 therapy within three individual response groups; PD patients (*n* = 9), SD patients (*n* = 9), and CR/PR patients (*n* = 3). Results shown include only the changes deemed statistically significant (*P* ≤ 0.05)

Significantly elevated in CRs/PRs			
Cytokine	Response	Δ Post-Pre(pg/mL)	*P*
GMCSF	CR/PR	1.45	0.05
**Significantly changed in SD**			
**Cytokine**	**Response**	**Δ Post-Pre (pg/mL)**	***P***
IL10	SD	3.63	0.02
VEGF-C	SD	−103.31	0.05
**Significantly elevated in PD**			
**Cytokine**	**Response**	**Δ Post-Pre (pg/mL)**	***P***
CCL3	PD	105.42	0.0009
CCL4	PD	341.97	0.001
IL18	PD	2,414.08	0.003
TNFα	PD	7.60	0.004
CXCL10	PD	18,152.00	0.005
CCL8	PD	269.25	0.01
IL10	PD	3.75	0.02
CCL2	PD	293.33	0.03
IL6	PD	5.09	0.03
IFNγ	PD	2,113.88	0.04

### Post-IL12 Increases in CCL3, CCL4, and IL6 were Significantly Higher in Patients with PD Compared with Those That Experienced CB

**FIGURE 3:**
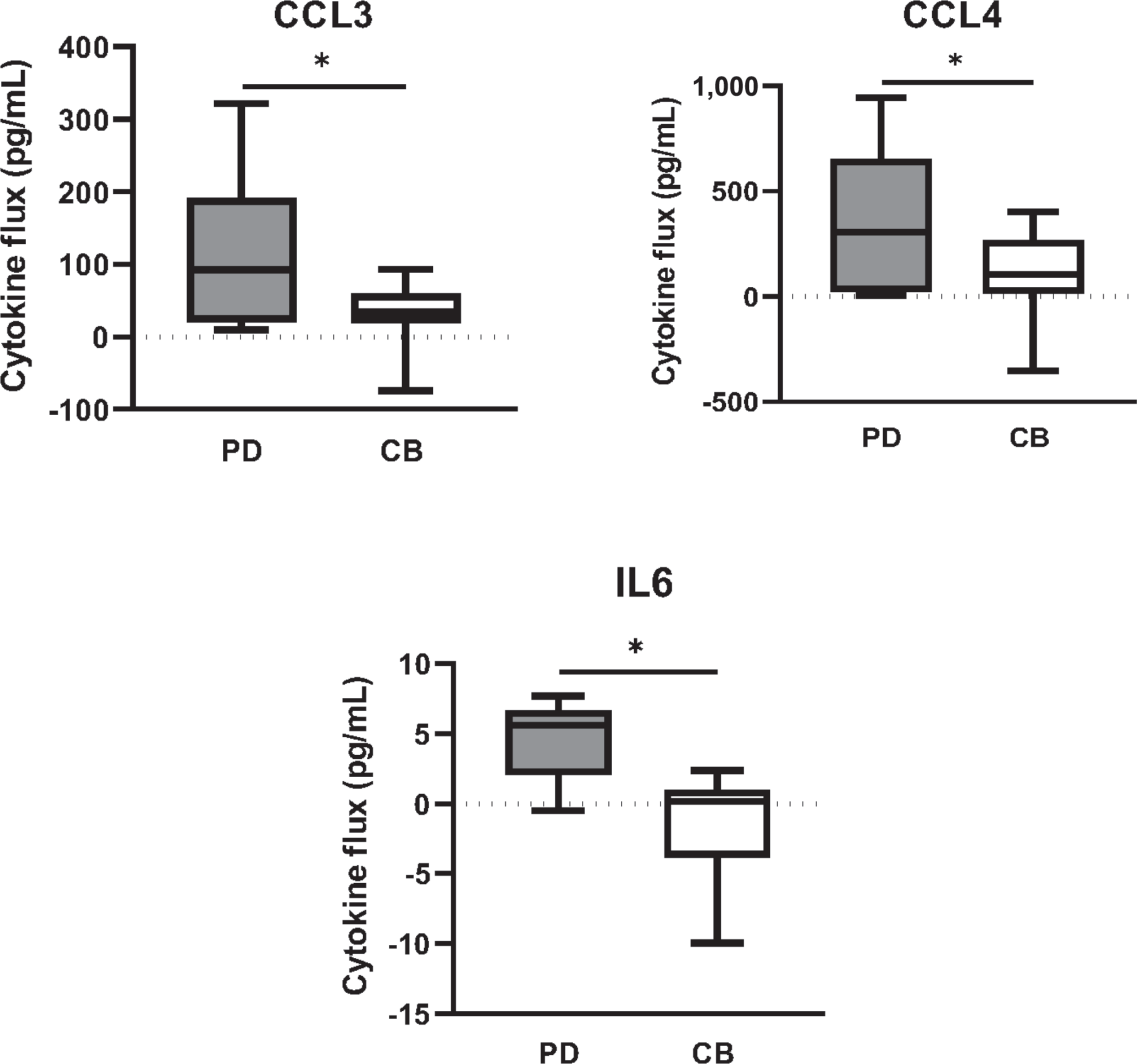
Cytokine fluxes according to patient response group. Changes in each cytokine level (pg/mL) following IL12 therapy were calculated compared with baseline. These changes were then compared between patients with PD and patients experiencing CB. CB is defined as SD, PR, or CR. ANOVA was employed to test differences in cytokine flux between patient response groups. Cytokines shown here were found to have significantly increased changes in PD patients compared with clinically benefitting patients following IL12 therapy. ANOVA, *, *P* < 0.05.

We next sought to determine whether the changes in cytokine and chemokine levels within each response group could be used as potential biomarkers of response. Using ANOVA analyses, we examined whether there were statistical differences in cytokine fluxes posttreatment in PD patients compared with those that experienced CB (SD, PR, or CR). We found that increases in circulating *CCL3, CCL4,* and IL6 levels post-IL12 were significantly higher in PD patients than clinically benefitting patients (*P* = 0.03 for all three cytokines; Fig. 3). On average, PD patients had an 80.24 pg/mL greater increase in CCL3 levels than clinically benefitting patients, a 274.9 pg/mL higher increase in CCL4 and a 6.04 pg/mL higher increase in IL6 following IL12 treatment. The post-IL12 increase in CCL3 and CCL4 levels were also higher in PD patients than both SD and PRs/CRs individually; however, this was not significant (*P* = 0.089, *P* = 0.097, respectively; Supplementary Fig. S1). The variability in the magnitude of these individual changes may be attributed to the physiologic role of each individual cytokine, their cellular source as well as their degree of induction by IL12. In addition, due to limited sample availability, levels of IL6 were only measured in patients with PD and SD, excluding CR/PR patients from comparison.

### Increases in CCL3, CCL4, and IL6 Occurred within the First Two Cycles of IL12 Therapy

**FIGURE 4:**
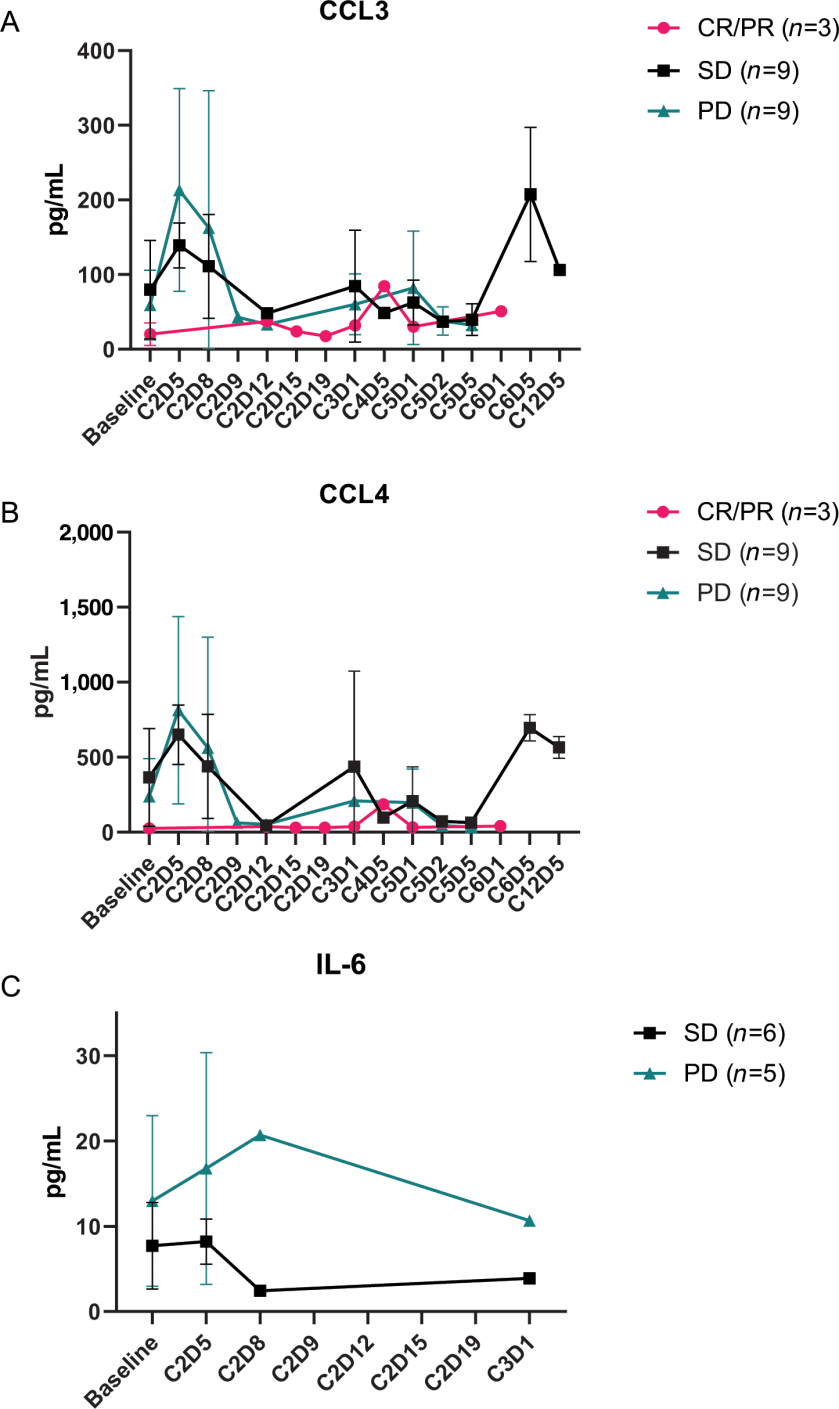
Extended timelines showing fluctuations in differentially expressed cytokine levels according to patient response. Cytokine levels of CCL3 (**A**), CCL4(**B**), and IL6 (pg/mL; **C**), collected 72 hours after IL12 administration, were measured at additional timepoints through cycle 3 for IL6 and cycle 12 for CCL3, and CCL4. Averages from each patient response group at the additional timepoints are plotted as individual points along the curves. Patient response groups are represented by PD patients, SD patients, and CR/PR patients.

Plasma from additional timepoints following IL12 administration were analyzed to characterize changes in CCL3, CCL4, and IL6 over time. Levels of CCL3 and CCL4 post-IL12 administration were measured at 13 additional timepoints and IL6 levels were measured at six additional timepoints (Fig. 4). Plasma samples from OSU-11010 extended through cycle 12 for CCL3, CCL4, and IL6, while samples from OSU-9968 extended through cycle 6 for CCL3 and CCL4 and cycle 3 for IL6. OSU-0167 samples extended through cycle 2 for both CCL3 and CCL4 but limited sample availability prevented their analysis for IL6 levels as well (Supplementary Fig. S2). Patterns of cytokine fluctuation varied distinctly within each of the three response groups for all three cytokines. Levels of CCL3 peaked in PD patients at C2D5 and declined rapidly by C2D9 (Fig. 4A). Levels remained low in this response group over the course of treatment. SD patients had a significantly lower increase in CCL3 levels at C2D5 (*P* = 0.03) but had a pronounced increase at C6D5. CRs/PRs maintained low CCL3 levels throughout treatment with only a small increase at C4D5. Patterns of fluctuation in CCL4 levels followed similar trends to CCL3 (Fig. 4B). Similar to CCL3, CR/PR patients maintained low CCL4 throughout treatment. Levels of IL6 were only available for analysis in progressive and SD patients, but still, unique fluctuation patterns were observed (Fig. 4C). The increase in IL6 levels found in PD patients at C2D5 was significantly higher than that found in SD patients (*P* = 0.03).

### Baseline Levels of IL10 Were Significantly Lower in CR/PR Patients

**FIGURE 5:**
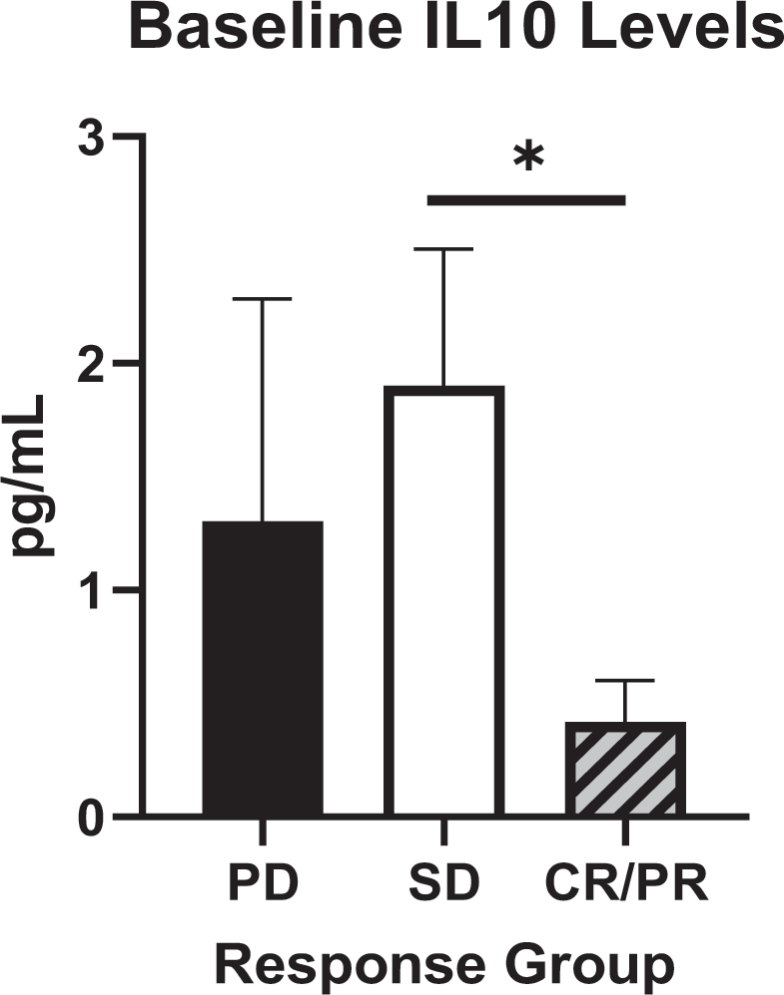
Baseline plasma levels of IL10 in PD, SD, and CR/PR patients. Plasma levels of IL10 (pg/mL) at baseline were measured in all patients and then analyzed according to post-therapy patient responses. Patient responses were classified into three groups; PD, SD, and CR/PR. Baseline levels were compared between the groups using *t* tests with *post hoc* Bonferroni corrections to detect any significant differences correlating with patient response. *P* values were deemed significant if *, *P* < 0.05.

Baseline cytokine levels were also analyzed to explore whether any pretreatment cytokine levels correlated with posttreatment patient responses. Of the 23 cytokines measured, only IL10 levels were significantly different at baseline depending on patient response to therapy (Fig. 5). IL10 levels in CR or PR patients (0.42 ± 0.18 pg/mL) were on average lower than in SD patients (1.90 ± 0.60 pg/mL, *P* = 0.05) and in PD patients (1.30 ± 0.98 pg/mL, *P* = 0.25).

## Discussion

A pilot analysis of cytokine/chemokine profiles in a subset of patients from three IL12 clinical trials was conducted in an exploratory effort to investigate whether any of these analytes could be correlated with patient response. Plasma samples from 21 patients collected throughout treatment were measured for levels of 23 different cytokine and chemokines at baseline and 72-hour post-IL12 treatment. While it was found that 11 cytokines/chemokines were significantly induced in patients after IL12 treatment, patterns and trajectories of cytokine fluctuations were unique to each patient response group. CR/PR patients had significant induction of GMCSF, while SD patients had a significant increase in IL10 levels and a significant decrease in VEGF-C levels. PD patients had increases in nine cytokines/chemokines including chemokines related to immune cell migration (CCL3, CCL4, CCL8, CCL2) and cytokines related to immune response (IL18, TNFα, CXCL10, IL6, IFNγ). Notably, increases in CCL3, CCL4, and IL6 in PD patients were significantly higher than clinically benefitting patients and occurred within the first two cycles of IL12 therapy. Cytokine/chemokine profiles at baseline also differed between patient response groups with IL10 levels being the lowest in CR/PR patients. Collectively, these results suggest that IL12 is capable of inducing changes in circulating cytokine/chemokine profiles that could potentially correlate with patient response. However, due to the heterogeneity of the patient populations and treatment regimens being evaluated, additional studies in larger cohorts of patients will be required to validate these results.

The differential induction of cytokines/chemokines in each patient response group suggests the potential for circulating cytokines to serve as an indicator of IL12 actions at the tumor level. An analysis of changes within each individual patient response group 72 hours after IL12 administration revealed that the only cytokine significantly upregulated in CR/PR patients after IL12 was GMCSF. GMCSF can be produced by many cell types in response to IL12 including NK cells, T cells, and macrophages (18). GMCSF has also been shown to act in a synergistic manner with IL12 to promote increased antitumor immune responses in melanoma, hepatocellular carcinoma, and sarcoma (19–22). This IL12/GMCSF antitumor effect was mediated predominantly by CD4^+^ T cells and IFNγ induction (22). These findings indicate that IL12 could activate both NK cell and T-cell immune responses. In addition, SD patients had significant increases in IL10 levels and significant decreases in VEGF-C levels. IL10 is a negative regulator of IL12 and could be increased in SD patients as a feedback mechanism following IL12-induced immune activation (23). It has also been shown that IL12-induced IL10 production is strongly mediated by CD4^+^ T cells and suggests once again that a clinically beneficial response to IL12 might rely on this T-cell population (23, 24). VEGF-C, on the other hand, is an angiogenic factor which has been shown to structurally and functionally alter tumor vasculature and enhance metastatic potential (25). IL12 has been shown to suppress VEGF effects in multiple tumor types through IFNγ and NK cell–mediated mechanisms (26). The significant decrease in VEGF-C in SD patients may indicate another beneficial response mechanism to IL12. In contrast to the clinically benefitting patients, PD patients had nine cytokines/chemokines significantly upregulated in response to IL12. However, these included many cytokines/chemokines (CCL3, CCL4, CCL8, CCL2, and IL6) shown to promote migration and accumulation of immunosuppressive myeloid populations such as myeloid-derived suppressor cells (MDSC) and tumor-associated macrophages (TAM; refs. 27, 28). These immunosuppressive cells may therefore represent a key mechanism of resistance to IL12 therapy as both MDSC and TAMs have been shown by our group to inhibit NK cells and T-cell activity (15, 28, 29).

In line with the possibility that increased immunosuppression may be driving IL12 resistance, analysis of cytokine/chemokine flux between response groups revealed significantly higher increases in CCL3, CCL4, and IL6 in PD patients than in CB patients. Increases in CCL3 and CCL4 in PD patients were also higher than in CR/PR patients, but the reduced number of patients in this group prevented the results from being statistically significant. CCL3 and CCL4 can promote migration of immunosuppressive MDSC and TAMs into the tumor microenvironment (TME) and have been shown to correlate with worsening patient outcomes (30, 31). In addition, blockade of the CCL3/CCL4 receptor CCR5 has been shown to suppress tumor growth and myeloid cell infiltration in a murine model of breast cancer (32). CCL3 and CCL4 can also be produced by MDSC and TAMs themselves, leading to both autocrine and paracrine STAT3 activation and subsequent enhancement of cancer cell proliferation, invasion, and epithelial–mesenchymal transition (33, 34). Similarly, intratumoral and circulating IL6 levels have been shown to correlate with increased tumor progression, worsening patient outcomes and the accumulation and activation of both MDSC and TAMs (27, 35, 36). Notably, IL6 has also been shown to mediate solid tumor resistance to both chemotherapy and immunotherapy by promoting the accumulation of systemic and intratumoral CD11b^+^ myeloid cells with impaired antigen presentation and maturation (37, 38). Thus, the significant increase in these three cytokines/chemokines may be indicative of an increasingly immunosuppressive TME unique to PD patients. Given the well-documented ability of MDSC and TAMs to suppress antitumor immunity, it is plausible that immunosuppression driven by increased MDSC and TAMs may be preventing beneficial patient responses to IL12 therapy (29, 39, 40). In addition, though increases in some cytokines commonly associated with a positive immune response to IL12 (IFNγ, IL18, CXCL10) were found in patients with PD, we posit the simultaneous increase in cytokines associated with increased immunosuppression (CCL3, CCL4, IL6) may have suppressed a beneficial patient response. This is supported by our findings that only PD patients had significant increases in these potentially suppressive cytokines.

The prognostic significance of circulating cytokine and chemokine levels has been demonstrated in a variety of cancers and provides support for their use as biomarkers (17, 41). Because of the central role that cytokines and chemokines play in mediating antitumor immune responses, it is not surprising that their levels can be vital indicators of how patients are responding to therapy (42, 43). However, it must be noted that using circulating analytes decreases our ability to make definitive conclusions about which specific cell type is producing or responding to each cytokine. Another limitation of this analysis is the relatively small sample size representing each patient response group. Sample sizes were small primarily due to the fact that many of the samples collected from these studies were used in generating other publications reporting respective clinical trial results and analyses (14–16). In addition, IL12 administration, like many immune-based therapies, does not always generate a large number of CRs or PRs. Importantly, despite these issues, we identified patterns within each of the three response groups that reached statistical significance. We also emphasize that this is a correlative pilot study from which definitive conclusions cannot be made; however, we believe these findings do support the continued investigation into this topic. Furthermore, the studies analyzed here utilized multiple treatment schemas; however, all three studies were similar in that they all employed a mAb backbone. The thinking in combining the studies for analysis was that any common signal would have clinical significance. To this end, we were able to find significant changes in cytokine/chemokine levels that remained consistent across the three studies and were concordant with the literature regarding the effect of IL12 on the immune system. The fact that these three separate studies showed similar effects of IL12 indicates the potential robustness of the data. In line with this, although multiple tumor types were included in this study, patients with similar clinical outcomes exhibited consistent immunologic changes in response to IL12. This finding suggests that the host immune response to IL12 may represent an inducible event that occurs independently of host tumor type. Indeed, the finding of similar IL12-induced cytokine patterns in patients with multiple tumor types suggests the possible existence of a universal immune phenomenon. However, in the final analysis, it must be emphasized that this is a correlative pilot study from which definitive conclusions cannot be made. The findings presented here should be viewed as preliminary and supportive of continued investigation into this topic.

It must also be noted that we and others have characterized changes in cytokine/chemokine levels in patients following mAb or chemotherapeutic treatments. (44, 45) This opens the possibility that some of the changes in cytokine levels that were detected in patients may have resulted purely from the interaction of IL12 with these other therapies. We acknowledge this limitation of the study. However, for each study, baseline plasma samples were collected prior to the start of any treatment in each study and single-agent IL12 was given at least 24 hours following trastuzumab, cetuximab, or paclitaxel/trastuzumab administration. Moreover, in the article published from the OSU-0167 trial, it was reported that within any given cycle, plasma levels of IFNγ peaked only following injections of IL12 rather than following antibody or chemotherapy administration. (15) A clinical study published in 2004 also evaluated changes in plasma levels of cytokines following single-agent paclitaxel therapy and found only significant increases in IL8 (not seen here) and IL6, which were less than half of the increase we detected in PD patients (46). An additional study looking at cytokine changes following EGFR-targeted therapy found minimal changes in many cytokines 24 hours after therapy, including CCL3 and CCL4 (47). This suggests that the changes in cytokine levels found in this study are more likely a result of IL12 therapy. Thus, while other drugs may have been on board, their impact on the post-IL12 cytokine levels should be low. Given the current use pattern of IL12-based drugs in combination regimens, it is difficult to obtain samples when only IL12 is on board. However, we and others have shown that several cytokines seen in this study are known to be NK-cell generated following IL12 therapy and associated with clinical responses (14, 48). We have also recently published a review discussing biomarkers of response to IL12 therapy across the known studies that used IL12, and several of the cytokines found in the current study (IFNγ, TNFα, CCL3, VEGF, and CXCL9) were consistent with our literature review (17).

In conclusion, this study utilized plasma samples from a subset of patients from three IL12 clinical trials in an effort to identify common changes in cytokine/chemokine profiles potentially correlative with patient responses. Eleven cytokines/chemokines were found to be significantly induced in patients after IL12 therapy. Moreover, distinct changes in cytokine/chemokine profiles were detected within each patient response group. CR/PR patients had increases in GMCSF, as opposed to SD patients which had increases in IL10 and decreases in VEGF-C, and PD patients which had increases in CCL3, CCL4, IL18, TNFα, CXCL10, CCL8, CCL2, IL6, and IFNγ. Increases in CCL3, CCL4, and IL6 were also significantly higher in PD patients than those who experienced CB and occurred within the first two cycles of IL12 therapy. These significant increases in CCL3, CCL4, and IL6 detected early in PD patients may provide a mechanistic understanding of poor responses to IL12 therapy and create a potential opportunity for therapeutic improvement. In addition, our ability to noninvasively detect these unique changes within the first two cycles of therapy and correlate them with patient response represent a generalizable method. In summary, this series of exploratory correlative findings should be viewed as a starting point for further investigation, rather than a definitive set of biomarkers for predicting the response to IL12 therapy. Additional biomarker studies focusing on the impact of IL12 administration on cytokine/chemokine levels in more homogenous patient cohorts will be required to confirm these findings and provide insight into the mechanism of action for this cytokine.

## Supplementary Material

Figure S1 Difference in cytokine fluxes between progressive disease, stable disease and complete/partially responding patients.

Figure S2 Extended cytokine expression timelines according to clinical study.
