# Supplementary material for: Analysis of Changes in Plasma Cytokine Levels in Response to IL12 Therapy in Three Clinical Trials
Source: Cancer Res Commun. 2024 Jan 10;4(1):81–91. doi: 10.1158/2767-9764.CRC-23-0122 (PMC10777814; doi:10.1158/2767-9764.CRC-23-0122)
Supplement: Figure S1 — Difference in cytokine fluxes between progressive disease, stable disease and complete/partially responding patients. [file crc-23-0122-s01.pdf]

Figure S1

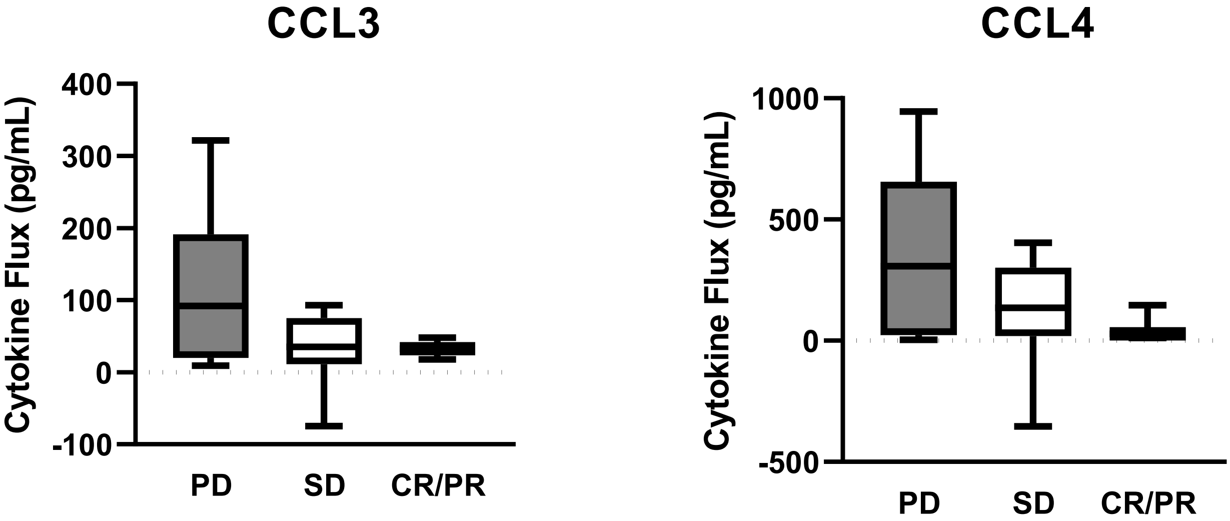

Fig. S1. Difference in cytokine fluxes between progressive disease, stable disease and complete/partially responding patients. Changes in each cytokine level (pg/mL) following IL-12 therapy were calculated compared to baseline. These changes were then compared between patients with progressive disease (PD), patients experiencing stable disease (SD), and patients with either partial responses or complete responses (CR/PR). ANOVA was employed to test differences in cytokine flux between response groups.
