# Supplementary material for: Analysis of Changes in Plasma Cytokine Levels in Response to IL12 Therapy in Three Clinical Trials
Source: Cancer Res Commun. 2024 Jan 10;4(1):81–91. doi: 10.1158/2767-9764.CRC-23-0122 (PMC10777814; doi:10.1158/2767-9764.CRC-23-0122)
Supplement: Figure S2 — Extended cytokine expression timelines according to clinical study. [file crc-23-0122-s02.pdf]

Figure S2

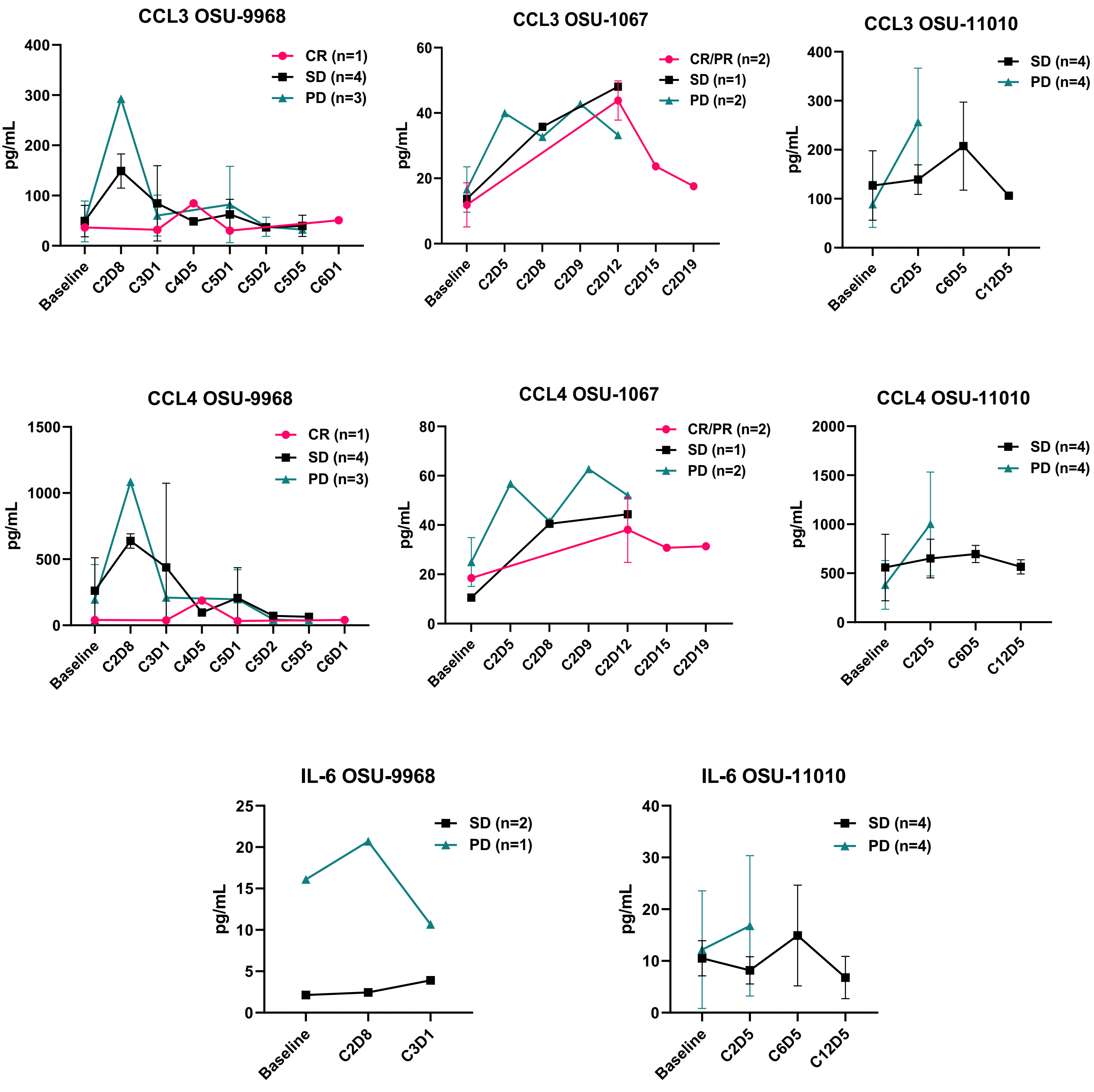

Fig. S2. Extended cytokine expression timelines according to clinical study. Levels of the CCL3, CCL4 and IL-6 (pg/mL) over time are plotted according to patient response and clinical study. Patient subsets are divided into progressive disease patients (PD), stable disease patients (SD) and complete/partially responding patients (CR/PR). Average cytokine levels from all patients within each response group at the included timepoints are plotted as individual points along the curves.
